# Relationships between abnormal neural activities and cognitive impairments in patients with drug-naive first-episode schizophrenia

**DOI:** 10.1186/s12888-020-02692-z

**Published:** 2020-06-05

**Authors:** Wei Yan, Rongrong Zhang, Min Zhou, Shuiping Lu, Wenmei Li, Shiping Xie, Ning Zhang

**Affiliations:** 1 Department of Psychiatry, Affiliated Nanjing Brain Hospital, Nanjing Medical University, Nanjing, 210029 China; 2 School of Geographic and Biologic Information, Nanjing University of Posts and Telecommunications, Nanjing, 210023 China; 3 College of Telecommunications & Information Engineering, Nanjing University of Posts and Telecommunications, Nanjing, 210003 China; 4 Smart Health Big Data Analysis and Location Services Engineering Lab of Jiangsu Province, Nanjing, 210023 China

**Keywords:** First-episode schizophrenia, Regional homogeneity, MCCB

## Abstract

**Background:**

Prior resting state functional Magnetic Resonance Imaging studies (rs-fMRI) via the regional homogeneity (ReHo) method have demonstrated inconsistent and conflicting results because of several confounding factors, such as small sample size, medicinal influence, and illness duration. Relationships between ReHo measures and cognitive impairments in patients with drug-naive First-Episode Schizophrenia (dn-FES) are rarely reported. This study was conducted to explore the correlations between ReHo measures and cognitive deficits and clinical symptoms in patients with dn-FES.

**Methods:**

A total of 69 patients with dn-FES and 74 healthy controls were recruited. MATRICS Consensus Cognitive Battery (MCCB), Wechsler Adult Intelligence Scale (WAIS), and Positive And Negative Syndrome Scale (PANSS) were used to assess cognitive function, Intelligence Quotient (IQ), and clinical symptoms, respectively. The correlations between ReHo maps and cognitive deficits and the severity of symptoms were examined using strict correlation analysis.

**Results:**

ReHo values in right Middle Frontal Gyrus (MFG) and Superior Frontal Gyrus (SFG) increased in dn-FES group, whereas ReHo values in right cuneus decreased. Correlation analysis showed that the ReHo values in right MFG positively correlated with attention/vigilance impairments, social cognition deficits, and the severity of clinical manifestations.

**Conclusions:**

These findings suggested that abnormal spontaneous activities in right MFG reflect illness severity and cognitive deficits, which also serve as a basis for establishing objective diagnostic markers and might be a clinical intervention target for treating patients with schizophrenia.

## Background

Schizophrenia (SZ) is a disabled mental illness that affects 0.5% of the China’s population [1]. SZ patients show deficits in a broad array of domains, including perception, attention, memory, processing speed, reasoning, problem solving, and social cognition [2]. Cognitive impairments are core features of SZ and have a substantial influence on patients’ psychosocial life. Cognitive impairments have also emerged as important targets of treatment-oriented research [3, 4].

The Measurement and Treatment Research to Improve Cognition in Schizophrenia (MATRICS) Consensus Cognitive Battery (MCCB) is a standard cognitive battery and contains ten tests that measure seven cognitive domains: speed of processing, attention/vigilance, working memory, verbal learning, visual learning, reasoning and problem solving, and social cognition [3, 4]. Patients with SZ show significant cognitive deficits across multiple cognitive domains assessed with the MCCB [5–9].

Resting state functional Magnetic Resonance Imaging (rs-fMRI) is a useful non-invasive neuroimaging technique for exploring local spontaneous neural activities and studying cognitive impairments. Regional homogeneity (ReHo) is a method employed in rs-fMRI and based on Kendall’s coefficient of concordance to measure the temporal synchronization of the time series of each voxel and compare it with that of its adjacent neighbors [10]. Patients with SZ exhibit increased ReHo values in Dorsolateral Prefrontal Cortex (DLPFC) [11–13], medial prefrontal cortex [14], angular gyrus [15], precentral gyrus [16], and superior temporal gyrus [13] and decreased ReHo values in the superior temporal gyrus [13–16], occipital lobes [12, 15, 17], parietal lobule [14, 16], precentral lobule [13, 14], and postcentral gyrus [13, 15, 16]. A recent study showed positive correlations between attention function and mean ReHo values in the left middle frontal gyrus, right inferior/ middle temporal gyrus, angular gyrus, and inferior/superior parietal lobe in healthy control group, but no significant correlations were found in SZ group [18].

ReHo studies on SZ have shown several promising findings, but such findings are inconsistent and conflicting. The reduced function of DLPFC plays a major role in pathophysiology of SZ and is associated with executive function deficits, response inhibition, and working memory errors [7, 19–21]. However, other brain areas, such as the superior temporal gyrus [13–16] and precentral lobule [14, 16], have yielded inconsistent and controversial results, and the relationships between abnormal brain activities and cognitive impairments have not been well investigated.

Numerous researchers attempted to map the neuronal pathophysiology in schizophrenia, but the results were often contradictory. The underlying etiology and pathophysiology of SZ remain unknown, and the following reasons should be considered: (1) existing studies have no adequate statistical power because of a small sample size; (2) in previous studies, the main threshold setting at a low or even incorrect level for multiple tests produced hundreds or even thousands of false positives to reflect true effects; this setting should be replicated in future studies, but strict correction should be performed to prevent type I errors [22–25]; (3) the heterogeneity of patients related to multiple episode courses, illness duration, and medical effect causes inconsistent and contradictory findings.

In the current study, we hypothesized that patients with SZ exhibiting common or distinct brain activities would reflect the severity of psychotic symptoms and cognitive deficits. This study intended to investigate the ReHo and its association with the systematic cognitive dysfunction between patients with drug-naive First-Episode Schizophrenia (dn-FES) and matching healthy controls (HCs). It also aimed to explore the associations between signal changes and clinical symptoms and to provide more revealing and consistent findings. In addition, it is another purpose of the study to ascertain which functional brain aberrations would reflect the clinical moderators including illness severity and cognitive deficits.

## Methods

### Participants

Eighty-seven patients who had first-episode schizophrenia in the past three years and were never medicated were recruited from Nanjing Brain Hospital from January 2017 to December 2018. Eighty-two healthy controls matched in terms of age, gender and ethnicity were recruited from the same area through advertising. The general inclusion criteria for two groups were right handed, aged 16–45, and able to understand survey instructions and execute cognitive tests.

Two qualified clinical psychologists conducted the Mini-International Neuropsychiatric Interview (M.I.N.I.) [26] to confirm diagnosis with the DSM-IV diagnostic criteria for schizophrenia and to exclude participants with a history of affective disorders, head trauma, and substance abuse. Healthy controls were also interviewed using the M.I.N.I. and excluded according to the same criteria adopted in screening patients as well as a major psychiatric disorder and family history of psychotic disorder. The study was approved by Nanjing Brain Hospital Ethics Committee. After comprehensively describing and explaining the study, a written informed consent was obtained from each participant.

### Neuropsychological and clinical assessments

The MCCB [27, 28] was used to assess cognitive function. The present study included nine tasks across seven cognitive domains, including speed of processing (Category Fluency, Trails A, Brief Assessment of Cognition in Schizophrenia Symbol Coding), attention/vigilance (Continuous Performance Test), working memory (Wechsler Memory Scale-III Spatial Span), verbal learning (Hopkins Verbal Learning Test–Revised), visual learning (Brief Visuospatial Memory Test–Revised), reasoning and problem solving (The Mazes test), and social cognition (Mayer–Salovey–Caruso Emotional Intelligence Test). We corrected the original score for age, gender, and education to obtain a *T* score to evaluate cognitive function, and high score showed a good performance. We adopted the Wechsler Adult Scale of Intelligence (WAIS) to test the Intelligence Quotient (IQ). Positive And Negative Syndrome Scale (PANSS) was used to assess symptoms of the patients [29], and the process was carried out by two experienced psychiatrists.

### Image data acquisition

MRI scanning was performed with a Siemens 3.0-T signal scanner, and a standard head coil padded with foam was used to reduce head motion and scanner noise. All of the participants lay in a supine position and were instructed to stay still, close their eyes, keep awake, and let their mind go blank [10, 30]. Three-dimensional T1-weighted sagittal images were acquired using a brain volume sequence with following parameters: repetition time (TR) = 2300 ms, echo time (TE) = 2.96 ms, inversion time = 900 ms, flip angle (FA) = 9°, field of view (FOV) = 256 mm × 256 mm, matrix = 256 × 256, slice thickness = 1 mm, 192 sagittal slices, and acquisition time = 554 s. The images were acquired using a gradient echo single-shot echo planar imaging sequence with the following parameters: TR/TE = 2500/30 ms, FOV = 224 mm × 224 mm, matrix = 64 × 64, FA = 90°, slice thickness = 3.5 mm, no gap, 37 interleaved transverse slices, 149 volumes, and acquisition time = 379 s.

### Data preprocessing and processing

Functional images were preprocessed with the Matlab2013b platform and the Data Processing Assistant for rs-fMRI (DPARSF4.4, advanced edition) [31]. Data were calculated in an original space warped by diffeomorphic anatomical registration through exponentiated Lie algebra (DARTEL). The first four volumes of the BOLD data were discarded for each subject to allow the signal to reach equilibrium. The remaining images were corrected for the acquisition time delay between slices. All of the subjects should have no > 2 mm maximum displacement in any plane, 2° of angular motion, and 0.2 mm mean frame-wise displacement [32]. Then, the images were spatially realigned to the first image of each dataset, and movement parameters were assessed for each subject and corrected using the Friston 24 approach [33]. Several nuisance covariates (global brain, white matter, and cerebrospinal fluid signals) were regressed. The datasets were band-pass filtered to reduce low-frequency drift and high-frequency physiological respiratory and cardiac noise (0.01 < *f* < 0.1 Hz). ReHo was calculated on a voxel-by-voxel basis by calculating Kendall’s coefficient of concordance on the basis of regional homogeneity hypothesis, which estimates similarity in the time series of a given voxel to its nearest 26 voxels [10]. Each subject’s value was divided by the mean value of their whole-brain ReHo to eliminate the whole-brain effect to the utmost extent. The standardized ReHo images were spatially smoothed with a Gaussian filter with a full width at half maximum (FWHM) of 4 mm. Finally, ReHo values were used for statistical analysis.

### Statistical analysis

Statistical analysis was conducted using SPSS software (version20.0). Demographic and clinical variables of dn-FES and healthy controls were compared using two-sample *t*-test for continuous variables and chi-squared for categorical variables. *F*-test was used for data analysis of cognitive domains after taking smoking as a covariate controlling.

The ReHo maps were compared between the two groups by using the threshold-free cluster enhancement (TFCE) method with family wise-error (FWE) correction for multiple comparisons. Age, gender, education, and probability of gray matter were treated as covariates, and the threshold for significance was *p* < 0.05 [34]. In the following correlation analysis, the resultant significant ReHo map was used as inclusion mask.

Patients with schizophrenia were under voxel-wise correlation analysis to explore the correlations between the ReHo maps and the PANSS (positive symptoms, negative symptoms, general, and all totals) and the correlations between the ReHo maps and the MCCB (speed of processing, verbal learning, working memory, reasoning/problem solving, visual learning, attention/vigilance, social cognition, and overall composite). In addition, age, gender, education, illness duration and the PANSS total score were placed in the model as covariates. The permutation-based nonparametric inference was undertaken with 5000 permutations, and significance level was thresholded for the correction of multiple comparisons by using a TFCE of 0.05.

## Results

### Demographic data outcome

Data from 26 subjects (18 patients and 8 HCs) were excluded because of excessive head movement, and the rest of 143 subjects (69 patients and 74 HCs) were used for analyzing. No significant differences were found between dn-FES and HC group in terms of age (*p* = 0.083) or gender (*p* = 0.142), whereas significant differences were observed in education (*p* = 0.004). The effect of smoking was examined by adding this variable to the analysis model as covariate. The results of demographic data are listed in Table 1.

**Table 1:** Demographic data, IQ, the MCCB scores, and clinical information in patients and health controls

	dn-FES (*n* = 69)	HC (*n* = 74)	Statistics	*p* Value
Age (years, mean ± SD)	24.22 ± 7.08	26.27 ± 6.97	*T* = 1.75	0.083
Sex (male/female)	50/19	45/29	*X*^*2*^ *=* 1.48	0.142
Education (years, mean ± SD)	13.23 ± 2.81	14.69 ± 3.10	*T* = 2.94	0.004
DUP (mouths, mean ± SD)	13.74 ± 11.76	NA	NA	NA
Smokers/Nonsmorkers	6/63	0/74	*X*^*2*^ = 6.72	0.01
PANSS				
Positive symptoms	24.42 ± 3.88	NA	NA	NA
Negative symptoms	17.58 ± 4.10	NA	NA	NA
General	42.19 ± 3.55	NA	NA	NA
All totals	84.19 ± 8.25	NA	NA	NA
Cognitive domains				
Speed of processing	35.42 ± 10.79	51.22 ± 8.46	*F* = 50.48	< 0.001
Verbal learning	35.51 ± 11.07	46.35 ± 9.78	*F* = 19.62	< 0.001
Working memory	34.22 ± 8.92	45.12 ± 7.10	*F* = 33.56	< 0.001
Reasoning/problem solving	42.90 ± 11.56	53.53 ± 8.17	*F* = 20.24	< 0.001
Visual learning	39.45 ± 11.91	50.55 ± 7.87	*F* = 23.17	< 0.001
Attention/vigilance	35.04 ± 11.49	48.03 ± 7.22	*F* = 36.14	< 0.001
Social cognition	32.68 ± 11.28	37.81 ± 9.34	*F* = 4.99	0.008
overall composite	28.07 ± 12.27	45.97 ± 8.13	*F* = 55.01	< 0.001
IQ	104.28 ± 11.81	115.55 ± 8.30	*F* = 50.48	< 0.001

dn-FES, drug-naive First-Episode Schizophrenia; HC, Healthy Control; DUP, Duration of Untreated Psychosis; NA, Not Applicable. *p* < 0.05 represents a significant difference. The effect of smoking was controlled as a covariate to the analysis in cognitive domains

### Group differences in ReHo

**Fig. 1:**
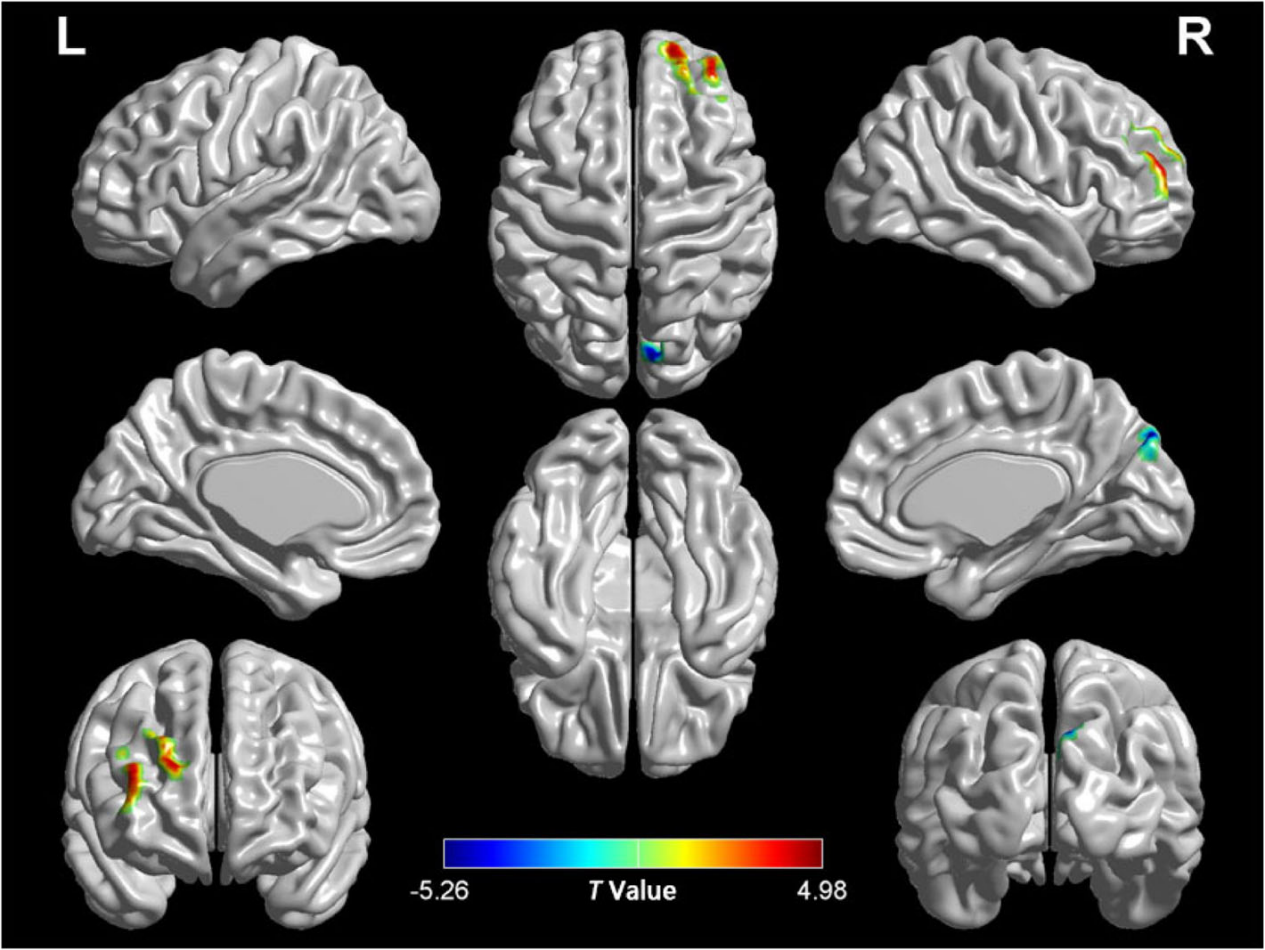
Brain regions with significantly altered ReHo between patients with dn-FES and HCs. Warm color indicated that ReHo was higher in the dn-FES group than in the HC group, and vice versa. The detailed *T* value was showed in Table 2. Statistically significant differences in gray matter volume were defined at *p* < 0.05. TFCE and FWE corrected after controlling age, gender, education, illness duration, and probability of gray matter

In this study, compared with the control group, the increased ReHo values were found mainly in right Middle Frontal Gyrus (MFG) and right Superior Frontal Gyrus (SFG), whereas the decreased ReHo values were observed in right cuneus (*p* < 0.05, TFCE, FWE corrected). Age, gender, illness duration, and probability of gray matter were corrected as covariates during calculation (Table 2, Fig. 1).

**Table 2:** Regions with ReHo differences in FES and HC subjects

	Brain regions	Brodmann area	Cluster	MNI coordinates (mm)			*T* Value
			(voxel)	X	Y	Z	
FES > HC	Right middle frontal gyrus	10	66	42	60	12	4.98
	Right superior frontal gyrus	9	37	27	45	42	4.44
FES < HC	Right cuneus	17	17	9	−84	39	−5.26

Statistically significant differences in ReHo between two groups were defined at *p* < 0.05. Data were TFCE and FWE corrected. Data were TFCE corrected after controlling age, gender, education, illness duration, and probability of gray matter

### Correlation analysis

Voxel-wise correlation analysis between the ReHo maps and the PANSS scores was performed to explore the relationships between the ReHo values and the PANSS scores in the dn-FES group by setting age, gender, education, and illness duration as covariates. Positive symptoms (*r* = 0.41, *p* < 0.05, corrected by TFCE), negative symptoms (*r* = 0.32, *p* < 0.05, corrected by TFCE), and the PANSS total scores (*r* = 0.45, *p* < 0.05, corrected by TFCE) had positive correlations with the ReHo values in the right MFG. The same approach was employed to investigate the relationships between the ReHo values and the MCCB in the dn-FES group. Attention/vigilance (*r* = − 0.30, *p* < 0.05, corrected by TFCE), social cognition (*r* = − 0.38, *p* < 0.05, corrected by TFCE), and overall composite (*r* = − 0.34, *p* < 0.05, corrected by TFCE) had negative correlations with the ReHo values in the right MFG (Tables 3 and 4).

**Table 3:** Significant correlations between the PANSS and ReHo in patients with schizophrenia

Brain regions	PANSS	Brodmann area	Cluster (voxel)	MNI coordinates (mm)			Correlation coefficient *r*
				X	Y	Z	
Right middle frontal gyrus	positive symptom	10	53	36	60	15	0.41
Right middle frontal gyrus	negative symptom	10	18	36	60	9	0.32
Right middle frontal gyrus	total score	10	42	36	60	12	0.45

Significant correlations between the PANSS and ReHo were at *p* < 0.05. Data were TFCE corrected after controlling age, gender, education, and illness duration as covariates

**Table 4:** Significant correlations between the MCCB subdomains and ReHo in patients with schizophrenia

Brain regions	MCCB	Brodmann area	Cluster (voxel)	MNI coordinates (mm)			Correlation coefficient *r*
				X	Y	Z	
Right middle frontal gyrus	Attention/vigilance	10	10	36	57	12	−0.30
Right middle frontal gyrus	Social cognition	10	15	39	57	9	−0.38
Right middle frontal gyrus	Overall composite	10	9	39	57	9	−0.34

Significant correlations between the MCCB subdomains and ReHo were defined at *p* < 0.05. Data were TFCE corrected after controlling age, gender, education, illness duration and the PANSS total score as covariates

## Discussion

In the present study, the dn-FES group showed severe and widespread cognitive deficits in all domains, and this observation was consistent with the previous studies that used the same instrument [5, 35, 36]. Schizophrenia has distinct cognitive impairments, including speed of processing [37], working memory [38], verbal learning [39], reasoning/problem solving [40], visual learning [41], attention/vigilance [42], and social cognition [43]. Our results showed that cognitive deficits were present at the onset of schizophrenia without considering the several factors, such as long-term antipsychotic treatment and prolonged illness course.

We observed increased ReHo values in right MFG (Brodmann Area 10, BA10) and SFG (BA9) and decreased ReHo values in right cuneus of patients with dn-FES as compared to HCs. The Prefrontal Cortex (PFC) is involved in the modulation of decision-making and executive control to behave appropriately by integrating sensory and emotional information [44, 45]. The frontal cortex lesion is related to behavioral impairments, such as inflexibility, social inappropriateness, isolation, and apathy [46]. The right MFG has been well studied and may play a major role in integrating visual, auditory, and somatic information to obtain an extensive and generalized interpretation of the environment [47]. Nevertheless, many research results are not entirely consistent and are even contradictory. For instance, some researchers have reported increased ReHo value in the frontal lobe [13, 48, 49], but others have failed to receive the same results and have even found decreased ReHo value in the frontal lobe [50]. Several factors, such as head motion regression model, cultural backgrounds, illness courses, illness heterogeneity, experimental parameters, and statistical methods, may be accountable. The cuneus belongs to visual cortex which is involved in visual processing and associated with attention and working memory. The abnormal regional spontaneous activities in the cuneus in the early stages of schizophrenia may cause higher-level cognitive impairments [51]. It was discovered that ReHo values decreased in right cuneus of patients with schizophrenia, which was partially consistent with previous findings [15, 49]. We proposed that the disruption of the local synchronization of the spontaneous activities in these brain areas be related to the psychopathology of schizophrenia. In particular, such common frontal aberrations may be concerned with vulnerability to psychosis.

The correlation analysis showed that ReHo values in right MFG positively correlated with the severity of clinical manifestations (positive symptoms, negative symptoms, and the PANSS total scores) in patients with dn-FES. These results were partially consistent with previous studies [48, 52, 53]. Decreased gray matter volumes in right MFG and SFG were associated with the total PANSS scores in patients with FES [53]. Neuroimaging studies have indicated that right MFG aberrations possibly play a crucial role in the emergence of psychotic symptoms of auditory hallucinations, thought disorder, and positive psychotic symptoms in schizophrenia [48]. Frontal lobe dysfunction possibly correlates with the occurrence of negative symptoms, especially in right MFG, which is particularly vulnerable to long-term effects of schizophrenia [52]. A partial brain area of right MFG overlapped the medial PFC, and a study on adolescent-onset schizophrenia has also shown that ReHo values in right medial PFC increased [14]. Another study has found that concomitant structural and causal connectivity deficits in medial PFC correlate with the negative symptoms of schizophrenia [54]. Similarly, our results suggested that negative symptoms positively correlated with ReHo values in right MFG in patients with dn-FES at the early stage. In summary, we found that ReHo values in right MFG were associated with not only positive symptoms but negative symptoms in dn-FES. The increased ReHo values in frontal regions were thought to underlie the manifestation of the psychotic symptoms in schizophrenia. However, considering that the data on the relationships between ReHo values and clinical variables in dn-FES are sparse, these findings may need further validation.

The correlation analysis showed that ReHo values in right MFG negatively correlated with the MCCB overall composite performance, social cognitive presentation, and attention/vigilance. A structural MRI study has found a thinner MFG cortex in patients with schizophrenia, and the decreased cortical thickness in this region is relevant to cognitive impairments [55]. This brain area has input and output connectivity with the supramodal cortex and functions as a mediator of information processing and information transfer during multiple cognitive operations [47]. The right MFG region is involved in higher cognitive functions, such as multitasking, planning of future actions, strategic processes in memory recall, various executive functions, and social cognition [56]. Moreover, ReHo values of right MFG negatively correlated with social cognition in our study. Social cognitive deficits in schizophrenia are broad, thereby imposing a huge burden on functional recovery. Severe social cognitive deficits exist in patients with schizophrenia, and social cognitive deficits are associated with abnormal spontaneous activities of the medial PFC [57]. The PFC is an important regulator in social cognition, and the disruption in prefrontal microcircuitry is thought to play an essential role in pathophysiology of schizophrenia with social deficits [45]. In this study, we found that ReHo values of right MFG negatively correlated with attention impairments. Attention deficits are core features of endophenotype of schizophrenia, which have yielded extensive researches [58, 59]. The right MFG is considered the connecting area of ventral and dorsal attention networks, which plays a prominent role in attention regulation [48].

Our study found increased ReHo values in right SFG. A meta-analysis also showed increased ReHo values in the SFG in both patients with first-episode schizophrenia and with treated schizophrenia [13]. The SFG is a significant component of the DLPFC [60]. The DLPFC is well-known for its involvement in executive functions, and in fact, it is also involved in risky and moral decision-making [61], working memory [62], and social cognition [63]. Abnormal neuronal activities in the DLPFC are associated with auditory hallucinations in schizophrenia [64–67] and other advanced cognitive functions [68]. However, in our study, ReHo values in the DLPFC did not significantly correlate with the MCCB and PANSS scores in the dn-FES group, which is affected by many factors, including the size and heterogeneity of samples and different indicators of spontaneous brain activities.

### Limitations

Current research has several limitations that should be improved in future research. First, the sample size is insufficient, although it is already more than that of most fMRI studies on first-episode schizophrenia to date, and our results could accept more stringent multiple comparison corrections. Second, we did not follow up these patients with schizophrenia, and hence, we were unable to determine the exact characteristics of brain changes and cognitive outcomes in schizophrenia and whether abnormal ReHo was a cause or a consequence of schizophrenia. Lastly, patients who could not keep their heads stable or complete all the cognitive tests were excluded from the study, which made the results unrepresentative to some extent.

## Conclusions

In conclusion, these findings indicate that increased ReHo values in right MFG and SFG are present even at the early stages of dn-FES, The frontal aberrations may be concerned with vulnerability to psychosis. The abnormal regional spontaneous neuronal activities revealed by ReHo values in right MFG are related not only to the negative and positive symptoms of schizophrenia, but also to attention and cognition deficits. These functional brain changes may reflect a clinical moderators including illness severity and cognitive deficits. This finding suggests that altered spontaneous brain activities in frontal cortex may reflect neuropathological characteristics of schizophrenia. Meanwhile, it serves as a basis for establishing objective diagnostic markers and clinical intervention target.

## Data Availability

The data sets used and /or analyzed during the present study are available from the corresponding author on reasonable request.

## Abbreviations

BA: Brodmann Area
DLPFC: Dorsolateral Prefrontal Cortex
dn-FES: Drug-naive First-Episode Schizophrenia
FWE: Family Wise-Error
MATRICS: Measurement and Treatment Research to Improve Cognition in Schizophrenia
MCCB: MATRICS Consensus Cognitive Battery
PFC: Prefrontal Cortex
MFG: Middle Frontal Gyrus
PANSS: Positive And Negative Syndrome Scale
ReHo: Regional Homogeneity
rs-fMRI: Resting state functional Magnetic Resonance Imaging
SFG: Superior Frontal Gyrus
TFCE: Threshold-Free Cluster Enhancement
WAIS: Wechsler Adult Intelligence Scale
